# Imaging and Circulating Biomarker-Defined Cardiac Pathology in Pulmonary Tuberculosis: A Systematic Review

**DOI:** 10.5334/gh.1369

**Published:** 2024-11-08

**Authors:** Marcello S. Scopazzini, Katherine J. Hill, Edith D. Majonga, Dominik Zenner, Helen Ayles, Anoop S. V. Shah

**Affiliations:** 1 Department of Non-Communicable Diseases Epidemiology, Faculty of Epidemiology and Population Health, London School of Hygiene and Tropical Medicine, Keppel Street, London, WC1E 7HT, United Kingdom; 2 Zambart, University of Zambia Ridgeway Campus, Off-Nationalist Road, Lusaka, Zambia; 3 Infection and Global Health Division, School of Medicine, Medical & Biological Sciences, University of St Andrews, St Andrews, KY16 9 AJ, United Kingdom; 4 Biomedical Research Training Institute, Harare, Zimbabwe; 5 University of Zimbabwe, Faculty of Medicine and Health Sciences, Department of Oncology, Medical Physics & Imaging Sciences, Harare, Zimbabwe; 6 Centre for Public Health and Policy, Wolfson Institute of Population Health, Queen Mary University of London, Mile End Road, London, E1 4 NS, United Kingdom; 7 Department of Clinical Research, Faculty of Infectious and Tropical Diseases, London School of Hygiene and Tropical Medicine, Keppel Street, London, WC1E 7HT, United Kingdom

**Keywords:** pulmonary tuberculosis, cardiovascular diseases, cardiac pathology

## Abstract

**Background:**

Pulmonary tuberculosis (PTB) is associated with increased cardiovascular disease (CVD) mortality. However, underlying pathophysiological mechanisms are poorly understood. This systematic review aims to synthesise the evidence on the prevalence of cardiac pathology based on cardiac imaging and circulating biomarkers in patients with PTB.

**Methods:**

We systematically searched databases for studies in patients with PTB evaluating cardiac pathology (pericardial effusion or left ventricular dysfunction) on echocardiography; late gadolinium enhancement on cardiac magnetic resonance imaging (CMR); myocardial inflammation on positron-emission tomography (PET); coronary artery stenosis on CT coronary angiography (CTCA); and cardiac troponin (cTn) and/or B-type natriuretic peptides (BNP) assessment.

**Results:**

Seven studies were included across 1,333 participants with PTB. Four studies used echocardiography (n = 1,111). The prevalence of pericardial effusion ranged from 14.1–55.9%; and left ventricular systolic impairment from 0–4.25%. One study used CMR and PET-CT (n = 26); and two studies used PET-CT alone (n = 196). The prevalence of pericardial and/or myocardial inflammation ranged from 0.6–21.8%. One study evaluated cTn, Creatine Kinase-MB (CK-MB), and BNP (n = 800), of whom 246 had raised cTn. No study reported cardiac pathology using CTCA.

**Conclusion:**

Pericardial effusion is the commonest reported cardiac pathology in PTB. To date, only one study has evaluated cardiac biomarkers and studies evaluating myocardial or coronary disease on advanced imaging remain limited. Our study highlights the paucity of evidence on the presence of cardiac pathology in PTB. Studies are required to determine the prevalence of, and disease mechanisms associated with cardiac pathology among patients with PTB.

## Introduction

Tuberculosis (TB) remains the leading cause of death by infectious disease worldwide and is of particular importance in high-burden countries including those in sub-Saharan Africa (SSA). Sub-Saharan Africa is also beleaguered by high Human immunodeficiency virus (HIV) prevalence rates with the region hosting many of the World Health Organization (WHO) defined high-TB and TB-HIV burden countries (1). Over two thirds of all deaths globally are due to non-communicable diseases, and nearly half of these are due to cardiovascular pathology (2). Low- and middle-income countries contribute to 80% of all cardiovascular diseases globally (2,3,4), with many of these countries also considered high TB- burden regions (1). There is now growing recognition of the role that infective pathologies may play in contributing to overall CVD burden (5).

Tuberculosis is a multi-system disease that predominantly affects the lung (pulmonary TB or PTB), and PTB accounts for up to 83% of incident cases (6,7). The remaining disease burden—termed extra-pulmonary TB (EPTB)—can affect any anatomical site including the heart in up to 2% of cases (1,7). Cardiac manifestations in TB are believed to occur secondary to lymphatic spread, typically affecting the right side of the heart and the pericardium (8,9). Well-described cardiac manifestations such as TB pericarditis carry up to 40% mortality at six months even with adequate treatment and are thought to be the main driver for up to 50% of pericardial diseases in high-TB burden countries (8).

To date, studies exploring the prevalence of, and associations between, pericardial and myocardial pathology and PTB has not been systematically evaluated and evidence remains limited to case series and reports and reviews (8,9). Research exploring the link between PTB and cardiac pathology has focused on Group III pulmonary hypertension (PH), a syndrome affecting the right side of the heart arising from chronic respiratory disease. In their comprehensive systematic review, van Heerden et al. reported a prevalence of PH of 9.4% in patients with active TB, rising up to 67% among patients with established post-TB lung disease (PTLD), and further studies are underway to better characterise the burden and mechanisms of PTB-induced PH (10,11,12).

Recent epidemiological data illustrates that globally, all-cause CVD mortality is up to 1.5-fold higher among patients with a history of PTB compared to those uninfected, but data are predominantly from high-income countries (HICs) with low TB endemicity (13). Post-mortem studies in Zambia report evidence of cardiac involvement at autopsy in 4–7% of TB deaths. This suggests that dissemination to cardiac structures is underappreciated, and mechanisms of cardiovascular disease associated with PTB remain poorly understood (14,15).

This systematic review aims to synthesise the evidence describing cardiac and vascular pathology in patients with PTB using 1) ultrasound and advanced cardiovascular imaging modalities and 2) circulating cardiac biomarkers of injury (cardiac troponin [cTn]) (16,17) and remodelling (brain natriuretic peptides [BNP]) (18) among patients with PTB.

## Methods

### Data sources, search strategy, and eligibility criteria

MEDLINE, Embase, Global Health, Web of Science, and Google Scholar were searched for studies evaluating cardiac pathology in patients with PTB using cardiac imaging techniques and/or biochemical markers of myocardial injury and remodelling.

The search strategy was performed using keywords for pulmonary tuberculosis (PTB), the imaging modalities, and cardiac biomarkers of injury and remodelling.

Our search terms included ‘pulmonary tuberculosis’ and related terms, ‘cardiac imaging’ including ‘echocardiography,’ ‘computed tomography coronary angiography,’ ‘cardiac magnetic resonance imaging,’ ‘positron-emission tomography,’ and ‘cardiac biomarkers’—see **Appendix 1 – Search Strategy** for full list of search criteria.

We included studies indexed from 1960 to October 31, 2023, and no restrictions were placed on language or study site.

Studies that used cardiac imaging and/or circulating biomarkers of cardiac injury and/or remodelling among patients with PTB were included. The primary exposure was bacteriological (microscopy, culture, or molecular diagnostics), radiological (chest x-ray or computed tomography), and/or clinical (according to internationally or locally accepted diagnostic algorithms) diagnosis of PTB.

### Study selection

**Figure 1:**
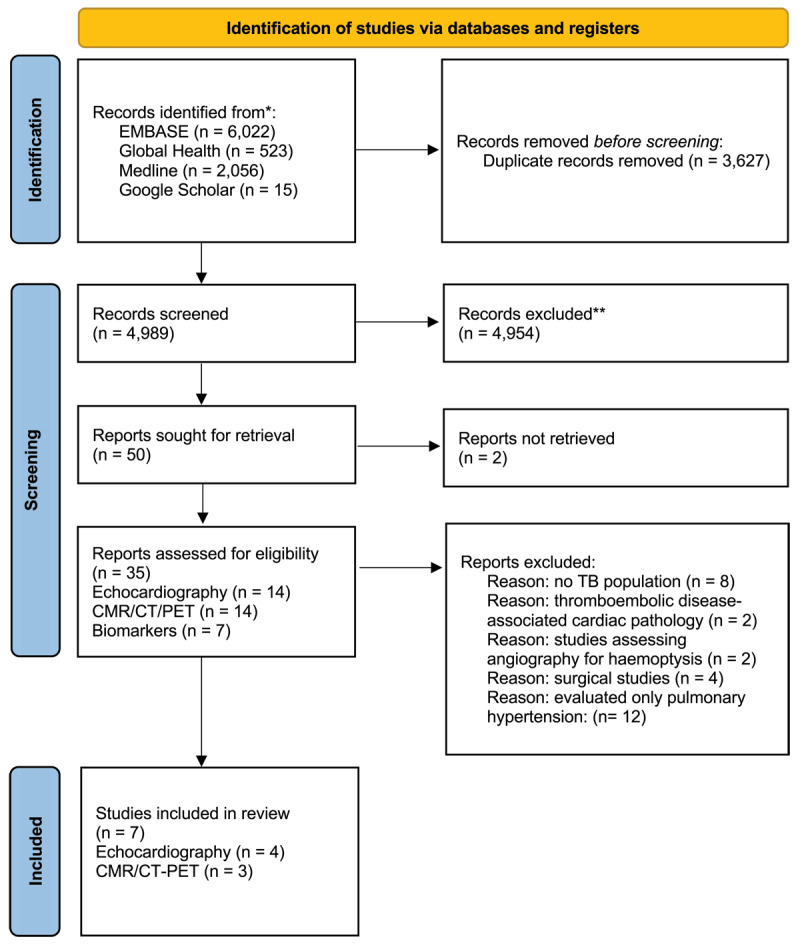
**Preferred Reporting Items For Systematic Reviews And Meta-Analyses (PRISMA) diagram.** Figure 1 describes our search strategy, including all relevant databases and subsequent reference checks employed in our study. We further describe our study selection and exclusion process culminating in our final seven included studies.

Two reviewers (MSS and KJH) screened titles and abstracts of retrieved studies; independently assessed their eligibility according to inclusion criteria; and resolved conflicts by consensus. A Preferred Reporting Items for Systematic Reviews and Meta-Analysis (PRISMA) chart was created (Figure 1). Data were extracted from studies by MSS and KJH and reviewed by ASVS.

### Outcomes

The main outcome was the proportion of patients with PTB who had evidence of cardiac pathology identified through cardiac imaging and/or biochemical markers of cardiac injury and remodelling.

Cardiac pathology was defined as: presence of pericardial pathology (pericardial effusion >0.5 cm) or left ventricular systolic dysfunction on echocardiography; myocardial fibrosis based on late gadolinium enhancement using CMR; coronary stenosis on CTCA; vascular and/or myocardial inflammation on PET; and myocardial injury based on circulating levels of cTn and BNP.

### Risk of bias

Risk of bias was independently assessed by two reviewers (MSS and KJH) using the National Heart, Lung and Blood Institute (NHLBI) quality assessment tools (19) for observational studies including cross-sectional, cohort, randomised controlled trial, and case series designs. These tools comprise 14 questions that assess the quality and risk of bias of studies and suggests inclusion, exclusion, or seeking further information prior to inclusion in the systematic review. Risk of bias was assessed primarily based on quality of study design.

## Results

### Study characteristics

We identified 4,989 studies after deduplication, of which seven met specified inclusion criteria (Figure 1 and Table 1) (20,21,22,23,24,25,26). A total of four studies evaluated echocardiographic findings of cardiac pathology: three studies (20,21,22) evaluated 533 participants using echocardiography alone; the other study (23) evaluated cardiac Troponin T (cTnT), Creatine Kinase-MB (CK-MB), and NT-pro-BNP alongside echocardiography in 800 participants. Two studies evaluated PET findings of vascular inflammation: one study (25) evaluated 358 patients with both pulmonary and extrapulmonary TB (PTB and EPTB), of whom 100 had pulmonary TB; the other study (24) evaluated 96 patients established on anti-tuberculous therapy. One study involving 26 patients described findings of lung and cardiac inflammation on PET and cardiac function on CMR (26). No study evaluated CTCA or transoesophageal echocardiography.

**Table 1:** **Study characteristics for seven included studies.** This table describes important features of each study included in this systematic review, including number of patients with PTB, the proportion of participants living with HIV, country of origin, method of TB diagnosis, and imaging method and outcome used to determine cardiac pathology. Imaging methods are: *TTE = transthoracic echocardiography; **Biomarkers = serum cardiac troponin T (cTnT), Creatine-Kinase MB (CK-MB), and non-terminal pro-B type natriuretic peptide (NT-pro-BNP); †CMR = cardiac magnetic resonance imaging; ‡FDG-PET = ^18^F-fluorodeoxyglucose positron emission tomography; §LGE = late gadolinium enhancement on CMR, a marker of myocardial inflammation; ||SUV max = maximum standardised uptake value for ^18^F-fluorodeoxyglucose positron emission tomography.


STUDY NAME	STUDY SUMMARY	STUDY PERIOD	STUDY DESIGN	COUNTRY	NUMBER OF PARTICIPANTS	CONTROL POPULATION	POPULATION SOURCE AND STUDY POPULATION	TB POPULATION (% OF TOTAL POPULATION)	PROPORTION OF PATIENTS LIVING WITH HIV (N (%))	TB DIAGNOSIS	OUTCOME MEASURE (IMAGING AND BIOMARKERS)

**Transthoracic Echocardiography (TTE) Studies**											

Casas, 2000	Cross-sectional study evaluating prevalence of pericardial effusion in patients with pulmonary TB (PTB)	1995–01 1997–12	Cross-sectional Study	Spain	85	No	Hospital inpatients—all consecutive new cases of PTB	100	36/85 (42.3%)	Microbiologically confirmed PTB	TTE^*^: Presence of pericardial effusion

Patel, 2010	Cross-sectional study evaluating the sensitivity and specificity of point-of-care ultrasound (POCUS) among patients with TB	2004–08 2004–10	Cross-sectional Study	South Africa	267	No	Hospital inpatients—consecutive patients with microbiologically confirmed PTB or EPTB	63.7	201/267 (75%)	Microbiologically confirmed PTB	TTE: Presence of pericardial effusion

Kahn, 2020	Prospective cohort study to evaluate the sensitivity and specificity of POCUS to detect PTB among patients presenting with two or more TB symptoms	2016–03 2017–08	Cohort Study	Malawi	181	Yes—participants without TB	Outpatients—all participants with two or more TB symptoms recruited consecutively	30.9	All PLHIV	Microbiologically confirmed PTB	TTE: Presence of pericardial effusion

Patil, 2023	Prospective follow-up study of consecutive patients newly diagnosed with PTB to describe features of cardiac dysfunction	2016–2020	Cohort Study	India	800	No	Respiratory outpatients—all patients with newly diagnosed pulmonary tuberculosis	100	0 (PLHIV excluded from study)	Microbiologically confirmed PTB	TTE: Global hypokinesia (visual assessment) Left ventricular systolic dysfunction Left ventricular diastolic dysfunction Biomarkers**: CK-MB, cTnT, and NT-pro-BNP levels (no cut-offs described)

**Cardiac Magnetic Resonance Imaging (CMR) and** ^18^**Fluorodeoxyglucose Positron-Emission-Tomography (FDG-PET) Studies**											

Mukasa, 2022	Nested cross-sectional study describing CMR^†^ and FDG-PET^‡^ features among patients enrolled to the StatinTB trial who recently completed TB treatment	Ongoing	Cross-sectional Study	South Africa	26	No	Patients enrolled in the StatinTB trial	100	6/26 23.1%	Microbiologically confirmed PTB	CMR^†^: Left ventricular end systolic volume FDG-PET^‡^: Persistent lung inflammation

Ankrah, 2019	Retrospective cross-sectional study describing radiological features on FDG-PET among patients established on anti-tuberculous therapy in Australia	Not stated	Cross-sectional Study	Australia	96	No	Radiology department—all cases on anti-tuberculous therapy included in the study	100	Not stated	Not stated	FDG-PET: myocardial SUV max^||^

Bomanji, 2020	Cross sectional study describing FDG-PET features among consecutively recruited patients with extrapulmonary tuberculosis (EPTB) in six different countries	Not stated	Cross-sectional Study	India, Pakistan, Thailand, Serbia, Bangladesh	358, of whom 100 had PTB	No	Hospital inpatients—all consecutive new diagnoses of extra-pulmonary tuberculosis	100	0 (PLHIV excluded from study)	Not stated	FDG-PET SUV max in all anatomical sites


Human immunodeficiency virus status was documented in six (20,21,22,23,25,26) of seven studies: one study (22) exclusively recruited People living with HIV (PLHIV); three studies (20,21,26) recruited both PLHIV and HIV-negative participants; one study (25) exclusively recruited participants with a documented negative HIV test; and one study (23) excluded PLHIV but did not provide HIV testing data. In the remaining study (24), participants’ HIV status was not reported.

**Figure 2:**
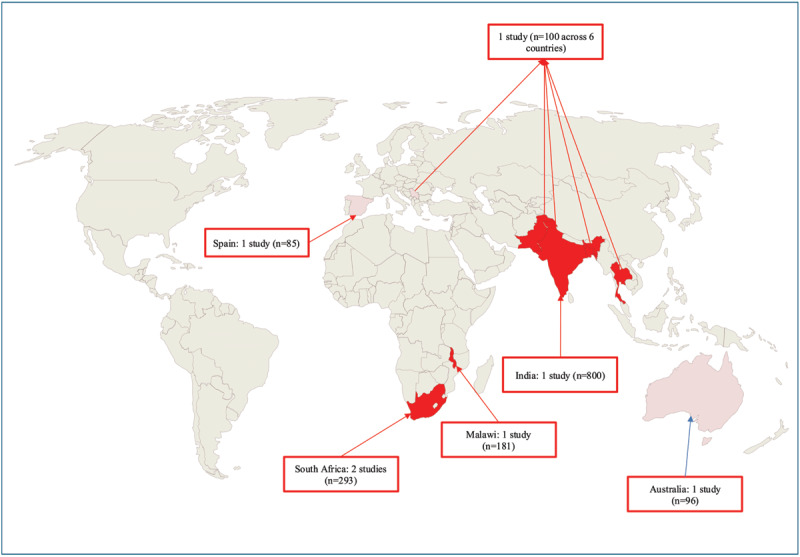
**Cartogram – geographical distribution of included studies by country TB endemicity.** This cartogram maps the geographical distribution of studies included in this systematic review. In dark red are countries defined as high-burden for tuberculosis incidence and prevalence as per World Health Organization; light pink indicates countries considered low-burden for tuberculosis. Arrows and boxes indicate the number of studies per country and number of participants with tuberculosis included in each relevant study.

Of the seven studies, four originated from high-TB burden countries (21,22,23,26) (one from South-East Asia, and three from sub-Saharan Africa), two from low-TB burden countries (20,24), and one (25) was a multi-country study with both high- and low-TB burden countries—**see** Figure 2.

The study designs used were cross-sectional (5/7, 71.4%) and cohort studies (2/7, 28.6%). Date of publication ranged from 2000 to 2023, and sample size ranged from 26 to 800 participants.

### Risk of bias assessment

Of the seven studies, three (42.9%) were classified as low (22,25,26); two medium (20,21) (28.6%); and two (23,24) (28.6%), high risk of bias. Of the included studies, three conducted statistical adjustment for confounders, none had adequate sample size justification, and one had a control population. Risk of bias assessment is described in **Appendix 2.**

### Population characteristics

A total of 1,333 participants, with PTB with a mean age ranging from 33 to 39.7 years of whom 243 (14.1%) were PLHIV and 56.3% were male, were enrolled across the studies (see Table 2 – **Population Characteristics**). Time since PTB diagnosis was available for all patients who were enrolled at the time of or shortly after PTB diagnosis.

**Table 2:** **Population characteristics of seven included studies.** This table describes the population characteristics of included studies and estimated prevalence rates of outcomes of interest: pericardial effusion; presence of left ventricular systolic dysfunction; and myocardial inflammation according to study-specific parameters. For Ankrah et al.: *patients with FDG SUVmax >10 consistent with cardiac inflammation on FDG-PET; for Bomanji et al.: †this was the prevalence of FDG SUV max uptake in pericardium consistent with pericardial inflammation; and for Mukasa et al. this was ‡Persistent lung inflammation defined as total lung glycolysis (TLG) >50 SUV/ml associated with left ventricular end systolic volume indicative of cardiac dysfunction.


SOURCE	NUMBER OF PATIENTS WITH PTB	MEN, %	WOMEN, %	AGE, MEAN, YEARS	TIMING OF IMAGING AND TB DIAGNOSIS	PARTICIPANTS LIVING WITH HIV, n/N, (%)	PRESENCE OF PERICARDIAL EFFUSION >0.5 CM, n/N, (%)	PRESENCE OF LEFT VENTRICULAR SYSTOLIC DYSFUNCTION, n/N, (%)	MYOCARDIAL INFLAM MATION, n/N, %	CARDIAC DYSFUNCTION ASSOCIATED WITH PERSISTENT LUNG INFLAMMATION, n/N, %

***Echocardiographic studies assessing pericardial effusion***										

Casas et al., 2000	85	72.9	27.1	39.7	Participants imaged at TB diagnosis	36/85(42.4)	12/85(14.1)	–	–	

Patel et al., 2010	170	NR	NR	36.4	Participants imaged at TB diagnosis	145/170(85.2)	95/170(55.9)	–	–	

Kahn et al., 2020	56	51.7	48.2	39	Participants imaged at TB diagnosis	56/56(100)	24/56(42.9)	–	–	

***Echocardiographic studies assessing left ventricular systolic function***										

Patil et al., 2023	800	56	44	NR	Participants imaged at TB diagnosis	0/800	–	34/800(4.25)	–	

***Positron emission tomography studies assessing vascular inflammation***										

Ankrah et al., 2019	96	49	51	37.4	67% of patients imaged within first two months of TB diagnosis	NR	–	–	21/96*(21.8)	

Bomanji et al., 2020	100	47	53	33	Participants imaged within two weeks of TB diagnosis	0	–	–	2/358†(0.6)	

***Studies evaluating both positron emission tomography and cardiac magnetic resonance imaging***										

Mukasa et al., 2022	26	61.5	38.5	37.8	Participants imaged at 24-week following TB diagnosis (completion of treatment)	6/26 (23.1)	NR	–		11/26‡ (42.3)


#### Echocardiography

A total of four studies (20,21,22,23) evaluated 1,111 patients with PTB using transthoracic echocardiography. Of these, three studies (20,21,22) with 311 participants evaluated the presence of pericardial effusion >0.5 cm in depth. The prevalence of pericardial effusion ranged from 14.1–55.9%. One study (23) evaluating 800 patients reported a prevalence of 4.25% for left ventricular systolic dysfunction.

Only one study (20) recruited participants with the explicit aim of determining incident pericardial effusion in patients with PTB. A further two studies (21,22) evaluated the diagnostic utility of point-of-care ultrasound among consecutively recruited patients with possible or probable PTB and reported prevalence estimates of pericardial effusion following confirmatory microbiological diagnosis.

#### Advanced cardiac imaging studies

One study (26), evaluating 26 patients who recently completed PTB treatment, described an association between reduced left ventricular end systolic volume (LVESV) on CMR and prevalence of persistent lung inflammation (PLI), defined as total lung glycolysis (TLG) >50 standardised uptake value (SUV) /ml FDG uptake on PET, in 11/26 (42.3%) participants.

One study (25) evaluated the diagnostic accuracy of FDG-PET in detecting EPTB in 358 patients across six different study sites, of whom 100 were confirmed to have dual PTB and EPTB. This study reported a 0.6% prevalence of perimyocardial FDG uptake consistent with pericardial involvement.

One study (24) of 96 participants established on anti-tuberculous therapy reported a 21.8% prevalence of myocardial inflammation defined as SUV >10.

#### Cardiac biomarkers

One study (23) evaluated serum cTnT, CK-MB, and NT-pro-BNP in 800 participants. The authors reported combined cTnT and CK-MB data but not NT-pro-BNP results in their manuscript. Of 208 participants with evidence of cardiac dysfunction on echocardiography, 98% (n = 198) had raised serum cTnT and/or CK-MB, compared to 7.7% (n = 592) of participants without echocardiographic evidence of cardiac dysfunction.

## Discussion

In this review, we aimed to synthesise the evidence for cardiac pathology (based on imaging and circulating biomarkers) among patients with PTB. Prevalence estimates for our outcomes of interest of any imaging-defined cardiac pathology demonstrated significant variation across studies with the most reported pathology being pericardial effusion and left ventricular systolic dysfunction. Only two studies (24,26) reported on myocardial pathology using advanced imaging, and only one study (23) attempted to correlate echocardiographic evidence of cardiac dysfunction with cardiac biomarkers. We found no studies that described associations between PTB and coronary stenosis.

Infection is a well-established precipitant of clinically evident acute and chronic cardiac pathology involving the pericardium (presenting as effusion and/or pericarditis); and myocardium (presenting as transient septic-induced cardiomyopathy (SIC), acute myocarditis, and/or chronic cardiomyopathies) (27,28,29,30,31). Indirect pathways linking infection to cardiac pathology involve systemic inflammatory responses. Immune cells upregulate pro-inflammatory cytokine production leading to oxidative stress and endothelial disruption associated with cardiac dysfunction. Echocardiography, advanced cardiac imaging, and cardiac biomarkers (16,18,32) reliably detect cardiac pathology and can plausibly indicate direct and indirect mechanistic associations between infection and clinical manifestations of cardiac pathology. Whilst these have been evaluated in other infective states such as HIV (33,34,35,36,37), their relevance to PTB—by far the commonest presentation of TB disease—remains unknown.

### Pericardial pathology in PTB

Three studies reported echocardiographic evidence of pericardial effusion in patients with PTB, with prevalence ranging from 14.1 to 55.6%. Across these studies, we made several observations. First, microbiological confirmation of tubercular effusion was not attempted and diagnosis of PTB-associated pericardial effusion was inferred from bacteriological confirmation in sputum. Importantly, one study reported a significant association between pericardial effusion and subsequent culture confirmation of TB in sputum (21). Second, all three studies were limited by sample size and cross-sectional designs. Third, the majority of participants were PLHIV, with only 4/131 patients with pericardial effusion and PTB who had a negative HIV test. Our evidence synthesis may therefore not reflect the true prevalence of pericardial effusion in patients with PTB—particularly in those who are HIV-negative—and it remains unclear whether PTB is associated with higher frequency of dissemination to the pericardium independent of co-existent HIV infection. Prevalence of pericardial effusions in other studies is restricted to populations with known cardiac pathology and TB disease. In these populations, and especially in those populations studied prior to the introduction of HIV antiretroviral therapy (ART), the reported prevalence of pericardial pathologies is up to 85% (38,39,40,41). Studies are now underway to systematically determine the prevalence and natural history of cardiac pathology, using cardiac ultrasound and biochemical markers, in patients with newly-diagnosed PTB, with and without HIV (42).

Tuberculosis is thought to contribute up to 50% of pericardial disease burden in LMICs (8) but systematic evaluation in the TB population remains sparse. Whereas bacterial pericarditis is almost uniformly fatal, TB pericarditis presents indolently with progressive accumulation of inflammatory serosal fluid and progressive calcification of inflamed pericardium (43). Despite its progressive symptomatology, TB pericarditis carries an up to 40% mortality at six months (43,44). The Investigation of the Management of Pericarditis (IMPI) trial (45) randomised patients with tuberculous pericarditis to adjuvant steroid therapy. Prednisolone administration resulted in a 40% reduction in development of constrictive pericarditis and hospitalization but a three-fold increase in HIV-associated cancer. Importantly, two-thirds of the study population were PLHIV and only 14% established on ART therapy.

### Myocardial pathology in PTB

Myocardial TB contributes <0.1% to incident TB cases, is associated with premature death from fatal arrhythmias, and diagnosed post-mortem in the few existing case studies (8,46). However, our study highlights the significant paucity of data to make firm conclusions on the interaction between PTB and myocardial pathology with data originating from a single study investigating an Indian population (23). One in 20 patients had depressed systolic function which was associated with biochemical evidence of cardiac injury. Importantly, in 20% of patients, left ventricular systolic dysfunction either persisted or worsened over time (23). Mechanistic imaging data using advanced modalities also remains limited. We identified one study that evaluated 26 patients with PTB using CMR and late gadolinium enhancement but did not report on the prevalence of myocardial fibrosis (26). Two further studies evaluated inflammatory cellular infiltration with PET in PTB (24,25) with a reported prevalence of perimyocardial inflammation at 0.6% and 21.8%. Myocardial TB may also be an unrecognised and important cause of significant cardiac electrical disturbance. A recent case series showed that of 13 patients presenting with unexplained ventricular tachycardia in India, 11 were found to have TB disease (47). Importantly, treatment with anti-TB medications and steroids resulted in improvement of left ventricular function and rhythm stability (47). Together, these findings further underline the need for systematic multi-modality imaging studies to delineate causal pathways in PTB-induced cardiac pathology at diagnosis, its potential impact on outcomes at TB treatment completion, and potential relationship with CVD burden in TB-endemic settings.

### Clinical correlations

Our evidence synthesis demonstrates the paucity of systematic evidence guiding healthcare providers to screen, diagnose, and manage cardiac involvement in PTB. Current data and clinical guidelines are overwhelmingly focused on a narrow definition of cardiac tuberculosis, overlooking clinically relevant cardiovascular complications that occur in patients with PTB.

Our review highlights significant heterogeneity in the available evidence describing cardiac pathology in PTB. It is plausible that the burden of cardiovascular pathology is underappreciated in PTB, and significant research gaps remain in evaluating the burden of cardiovascular pathology in PTB. Our review provides critical evidence to guide the next phase of research to understand the burden and mechanisms of TB-associated CVD.

## Limitations

Our systematic review has several important limitations. First, data describing imaging- and biomarker-defined cardiac pathology in patients with PTB are scarce and variability in the limited number of studies reporting on our outcomes of interest preclude meta-analysis. Second, studies in each outcome group demonstrated significant variability in patient population, study design, and covariate measurements, limiting our ability to amalgamate our findings. Cardiovascular risk factors were not reported in any of the studies, which limits our interpretation of cardiac pathology. Fourth, whilst we performed an advanced search of the grey literature including recent conference abstracts and posters, we may still have missed information relevant to our review. Fifth, we excluded case reports, studies with fewer than ten participants, and studies that did not evaluate cardiac imaging and/or biomarkers, limiting our ability to synthesise evidence of cardiac pathology derived from pathological studies.

## Conclusions

In this systematic review, we present summary data on the burden of imaging-defined cardiac pathology among patients with PTB. Whilst available data demonstrated significant heterogeneity precluding meta-analysis on outcomes of interest of cardiac imaging- and biomarker-defined pathology, our study suggests that cardiac pathology is underreported in patients with PTB. Systematic prevalence and observational cohort studies to better describe the burden and natural history of cardiac pathology at PTB diagnosis, during treatment, and post-treatment are needed in settings with high TB-endemicity.

## Data Accessibility Statement

Data are available under the terms of the Creative Commons Attribution 4.0 International License (CC-BY 4.0)

## Additional File

The additional file for this article can be found as follows:

10.5334/gh.1369.s1 Supplementary File. Appendix 1 and 2.

## Figures and Tables

**Table:** 

ART	antiretroviral therapy
BNP	brain natriuretic peptides
CKMB	creatine kinase MB
CMR	cardiac magnetic resonance imaging
CTCA	computed tomography coronary angiography
cTn	cardiac troponins
cTnT	cardiac troponin T
CVD	cardiovascular diseases
EPTB	extrapulmonary tuberculosis
FDG	fluorodeoxyglucose
HICs	high-income countries
HIV	human immunodeficiency virus
LGE	late gadolinium enhancement
LMICs	low- and middle-income countries
LVEF	left ventricular ejection fraction
LVESV	left ventricular end systolic volume
NT-pro-BNP	non-terminal pro-B type natriuretic peptide
PET	positron emission tomography
PH	pulmonary hypertension
PLHIV	people living with HIV
PLI	persistent lung inflammation
PTLD	post-TB lung disease
PTB	pulmonary tuberculosis
SIC	sepsis-induced cardiomyopathy
SSA	sub-Saharan Africa
SUV	standardised uptake value
TB	tuberculosis
TB-HIV	tuberculosis and HIV co-infection
TOE	transoesophageal echocardiography
TTE	transthoracic echocardiography
WHO	World Health Organization

## References

[1] World Health Organization. WHO global lists of high burden countries for tuberculosis (TB), TB/HIV and multidrug/rifampicin-resistant TB (MDR/RR-TB), 2021–2025: Background Document [Internet]. World Health Organization; 2021 [cited 2022 May 16]. Available from: https://apps.who.int/iris/handle/10665/341980.

[2] World Health Organization. Non communicable diseases [Internet]. World Health Organization; 2023 [cited 2023 March 15. Available from: https://www.who.int/news-room/fact-sheets/detail/noncommunicable-diseases.

[3] Mocumbi AO. Cardiovascular health care in low- and middle-income countries. Circulation. 2024; 149(8):557–559. DOI: 10.1161/CIRCULATIONAHA.123.065717 38377254

[4] Bennett JE, Stevens GA, Mathers CD, Bonita R, Rehm J, Kruk ME, et al. NCD Countdown 2030: Worldwide trends in non-communicable disease mortality and progress towards sustainable development goal target 3.4. Lancet. 2018; 392(10152):1072–1088. DOI: 10.1016/S0140-6736(18)31992-5 30264707

[5] Farina JM, Liblik K, Iomini P, Miranda-Arboleda AF, Saldarriaga C, Mendoza I, et al. Infections and cardiovascular disease: JACC focus seminar 1/4. J Am Coll Cardiol. 2023; 81(1):71–80. DOI: 10.1016/j.jacc.2022.08.813 36599613

[6] World Health Organization. Global tuberculosis report 2023 [Internet]. 1st ed. World Health Organization; 2023. [cited 2024 October 12]. Available from: https://iris.who.int/bitstream/handle/10665/373828/9789240083851-eng.pdf?sequence=1.

[7] Pai M, Behr MA, Dowdy D, Dheda K, Divangahi M, Boehme CC, et al. Tuberculosis. Nat Rev Dis Primer. 2016; 2(1):1–23. DOI: 10.1038/nrdp.2016.76 27784885

[8] López-López JP, Posada-Martínez EL, Saldarriaga C, Wyss F, Ponte-Negretti CI, Alexander B, et al. Tuberculosis and the heart. J Am Heart Assoc. 2021; 10(7):e019435. DOI: 10.1161/JAHA.120.019435 33733808 PMC8174360

[9] Marcu DTM, Adam CA, Mitu F, Cumpat C, Onofrei VA, Zabara ML, et al. Cardiovascular involvement in tuberculosis: from pathophysiology to diagnosis and complications—A narrative review. Diagnostics. 2023; 13(3). DOI: 10.3390/diagnostics13030432 PMC9914020 36766543

[10] van Heerden JK, Louw EH, Thienemann F, Engel ME, Allwood BW. The prevalence of pulmonary hypertension in post-tuberculosis and active tuberculosis populations: a systematic review and meta-analysis. Eur Respir Rev. 2024; 33(171):230154. DOI: 10.1183/16000617.0154-2023 38232991 PMC10792440

[11] Allwood BW, Manie S, Stolbrink M, Hunter L, Mathee S, Meintjes G, et al. Pulmonary hypertension in adults completing tuberculosis treatment. Afr J Thorac Crit Care Med. 2023; 29(3). DOI: 10.7196/AJTCCM.2023.v29i3.676 PMC10642409 37970573

[12] Louw E, Baines N, Maarman G, Osman M, Sigwadhi L, Irusen E, et al. The prevalence of pulmonary hypertension after successful tuberculosis treatment in a community sample of adult patients. Pulm Circ. 2023; 13(1):e12184. DOI: 10.1002/pul2.12184 36699148 PMC9852678

[13] Basham CA, Smith SJ, Romanowski K, Johnston JC. Cardiovascular morbidity and mortality among persons diagnosed with tuberculosis: A systematic review and meta-analysis. PloS One. 2020; 15(7):e0235821. DOI: 10.1371/journal.pone.0235821 32649721 PMC7351210

[14] Bates M, Mudenda V, Shibemba A, Kaluwaji J, Tembo J, Kabwe M, et al. Burden of tuberculosis at post mortem in inpatients at a tertiary referral centre in sub-Saharan Africa: A prospective descriptive autopsy study. Lancet Infect Dis. 2015; 15(5):544–551. DOI: 10.1016/S1473-3099(15)70058-7 25765217

[15] Mucheleng’anga LA, Himwaze CM, Telendiy V, Simumba S, Soko J, Kayonde N, et al. Incidental Tuberculosis in sudden, unexpected, and violent deaths in the community Lusaka, Zambia – A descriptive forensic post-mortem examination study. Int J Infect Dis. 2022; 124:S75–S81. DOI: 10.1016/j.ijid.2022.03.005 35283296

[16] Shah ASV, Anand A, Strachan FE, Ferry AV, Lee KK, Chapman AR, et al. High-sensitivity troponin in the evaluation of patients with suspected acute coronary syndrome: a stepped-wedge, cluster-randomised controlled trial. Lancet. 2018; 392(10151):919–928. DOI: 10.1016/S0140-6736(18)31923-8 30170853 PMC6137538

[17] Higgins JP, Higgins JA. Elevation of cardiac troponin I indicates more than myocardial ischemia. Clin Exp Med. 2003; 26(3):133–147. 12858947

[18] Welsh P, Campbell RT, Mooney L, Kimenai DM, Hayward C, Campbell A, et al. Reference Ranges for NT-proBNP (N-Terminal Pro-B-Type Natriuretic Peptide) and Risk Factors for Higher NT-proBNP Concentrations in a Large General Population Cohort. Circ Heart Fail. 2022; 15(10):e009427. DOI: 10.1161/CIRCHEARTFAILURE.121.009427 36098049 PMC9561238

[19] National Heart, Lung, and Blood Institute. Study Quality Assessment Tools [Internet]. NIH [updatd 2021 July; cited 2023 March 21]. Available from: https://www.nhlbi.nih.gov/health-topics/study-quality-assessment-tools.

[20] Casas E, Blanco JR, Ibarra V, Metola L, Rosel L, Oteo JA. Incidence of pericardial effusion in pulmonary tuberculosis. Int J Tuberc Lung Dis. 2000; 4(12):1173–1175. 11144461

[21] Patel MN, Beningfield S, Burch V. Abdominal and pericardial ultrasound in suspected extrapulmonary or disseminated tuberculosis. South Afr Med J. 2011; 101(1):39–42. DOI: 10.7196/SAMJ.4201 21626980

[22] Kahn D, Pool KL, Phiri L, Chibwana F, Schwab K, Longwe L, et al. Diagnostic utility and impact on clinical decision making of focused assessment with sonography for hiv-associated tuberculosis in Malawi: A prospective cohort study. Glob Health Sci Pract. 2020; 8(1):28–37. DOI: 10.9745/GHSP-D-19-00251 32041772 PMC7108937

[23] Patil SV, Toshniwal S, Acharya A, Gondhali G. Cardiac dysfunction in active pulmonary tuberculosis: Mysterious facts of TB’s pandora. Electron J Gen Med. 2023; 20(2):em452. DOI: 10.29333/ejgm/12834

[24] Ankrah A, Lawal I, Glaudemans A, Slart R, Sathekge M. Intense uniform FDG myocardial uptake during anti-tuberculous therapy. J Nucl Med. 2019; 60(supplement 1):1064.

[25] Bomanji J, Sharma R, Mittal BR, Gambhir S, Qureshy A, Begum SMF, et al. PET/CT features of extrapulmonary tuberculosis at first clinical presentation: A cross-sectional observational 18F-FDG imaging study across six countries. Eur Respir J. 2020; 55(2):1901959. DOI: 10.1183/13993003.01959-2019 31831584

[26] Mukasa S, Aremu O, Wolmarans K, Mashinyira A, Katoto P, Jakoet-Bassier F, et al. Comparing cardiac MRI and lung PET/CT after completion of tuberculosis treatment – preliminary findings of participants co-enrolled into the StatinTB trial and cardiac imaging after TB (CIA-TB) study. Eur Heart J. 2022; 43(Supplement_2):ehac544.2607. DOI: 10.1093/eurheartj/ehac544.2607

[27] Lazarou E, Tsioufis P, Vlachopoulos C, Tsioufis C, Lazaros G. Acute Pericarditis: Update. Curr Cardiol Rep. 2022; 24(8):905–913. DOI: 10.1007/s11886-022-01710-8 35595949 PMC9122084

[28] Imazio M, Cecchi E, Demichelis B, Chinaglia A, Ierna S, Demarie D, et al. Myopericarditis versus viral or idiopathic acute pericarditis. Heart. 2008; 94(4):498–501. DOI: 10.1136/hrt.2006.104067 17575329

[29] Imazio M, Brucato A, Maestroni S, Cumetti D, Belli R, Trinchero R, et al. Risk of constrictive pericarditis after acute pericarditis. Circulation. 2011; 124(11):1270–1275. DOI: 10.1161/CIRCULATIONAHA.111.018580 21844077

[30] Hollenberg SM, Singer M. Pathophysiology of sepsis-induced cardiomyopathy. Nat Rev Cardiol. 2021; 18(6):424–434. DOI: 10.1038/s41569-020-00492-2 33473203

[31] L’Heureux M, Sternberg M, Brath L, Turlington J, Kashiouris MG. Sepsis-Induced Cardiomyopathy: A comprehensive review. Curr Cardiol Rep. 2020; 22(5):35. DOI: 10.1007/s11886-020-01277-2 32377972 PMC7222131

[32] Rørth R, Jhund PS, Yilmaz MB, Kristensen SL, Welsh P, Desai AS, et al. Comparison of BNP and NT-proBNP in Patients with heart failure and reduced ejection fraction. Circ Heart Fail. 2020; 13(2):e006541. DOI: 10.1161/CIRCHEARTFAILURE.119.006541 32065760

[33] Hudson JA, Majonga ED, Ferrand RA, Perel P, Alam SR, Shah ASV. Association of HIV infection with cardiovascular pathology based on advanced cardiovascular imaging: a systematic review. JAMA. 2022; 328(10):951–962. DOI: 10.1001/jama.2022.15078 36098725 PMC9471974

[34] Ahmed HA, Mohamed J, Akuku IG, Lee KK, Alam SR, Perel P, et al. Cardiovascular risk factors and markers of myocardial injury and inflammation in people living with HIV in Nairobi, Kenya: A pilot cross-sectional study. BMJ Open. 2022; 12(6):e062352. DOI: 10.1136/bmjopen-2022-062352 PMC9171254 35667720

[35] Olalla J, Crespo E, De la Torre J, Sempere M, Del Arco A, Prada JL, et al. Factors related to NT-proBNP levels in HIV patients aged over 40 years. AIDS Res Ther. 2015; 12(101237921):17. DOI: 10.1186/s12981-015-0058-7 25960760 PMC4426165

[36] Peterson TE, Baker JV, Wong LY, Rupert A, Ntusi NAB, Esmail H, et al. Elevated N-terminal prohormone of brain natriuretic peptide among persons living with HIV in a South African peri-urban township. ESC Heart Fail. 2020; 7(5):3246–3251. DOI: 10.1002/ehf2.12849 32585776 PMC7524119

[37] Robbertse PPS, Doubell AF, Steyn J, Lombard CJ, Talle MA, Herbst PG. Altered cardiac structure and function in newly diagnosed people living with HIV: A prospective cardiovascular magnetic resonance study after the initiation of antiretroviral treatment. Int J Cardiovasc Imaging. 2023; 39(1):169–182. DOI: 10.1007/s10554-022-02711-y 36598696 PMC9412796

[38] Cegielski JP, Ramiya K, Lallinger GJ, Mtulia IA, Mbaga IM. Pericardial disease and human immunodeficiency virus in Dar es Salaam, Tanzania. Lancet. 1990; 335(8683):209–212. DOI: 10.1016/0140-6736(90)90288-G 1967676

[39] Maher D, Harries AD. Tuberculous pericardial effusion: A prospective clinical study in a low-resource setting—Blantyre, Malawi. Int J Tuberc Lung Dis. 1997; 1(4):358–364. 9432393

[40] Reuter H, Burgess LJ, Doubell AF. Epidemiology of Pericardial Effusions at a large academic hospital in South Africa. Epidemiol Infect. 2005; 133(3):393–399. DOI: 10.1017/S0950268804003577 15962545 PMC2870262

[41] Ntsekhe M, Hakim J. Impact of human immunodeficiency virus infection on cardiovascular disease in Africa. Circulation. 2005; 112(23):3602–3607. DOI: 10.1161/CIRCULATIONAHA.105.549220 16330702

[42] Scopazzini MS, Chansa P, Majonga ED, Bual N, Schaap A, Mateyo KJ, et al. The burden and natural history of cardiac pathology at TB diagnosis in a high-HIV prevalence district in Zambia: protocol for the TB-Heart study. BMC Cardiovasc Disord. 2024; 24(1):205. DOI: 10.1186/s12872-024-03877-0 38600454 PMC11007960

[43] Ntsekhe M, Mayosi BM. Tuberculous pericarditis with and without HIV. Heart Fail Rev. 2013; 18(3):367–373. DOI: 10.1007/s10741-012-9310-6 22427006

[44] Mayosi BM, Wiysonge CS, Ntsekhe M, Gumedze F, Volmink JA, Maartens G, et al. Mortality in patients treated for tuberculous pericarditis in sub-Saharan Africa: original article. S Afr Med J. 2008; 98(1):36–40. DOI: 10.10520/EJC69118 18270639

[45] Mayosi BM, Wiysonge CS, Ntsekhe M, Volmink JA, Gumedze F, Maartens G, et al. Clinical characteristics and initial management of patients with tuberculous pericarditis in the HIV era: the Investigation of the management of pericarditis in Africa (IMPI Africa) registry. BMC Infect Dis. 2006; 6(1):2. DOI: 10.1186/1471-2334-6-2 16396690 PMC1352368

[46] Michira BN, Alkizim FO, Matheka DM. Patterns and clinical manifestations of tuberculous myocarditis: A systematic review of cases. Pan Afr Med J. 2015; 21(118). DOI: 10.11604/pamj.2015.21.118.4282 PMC4546727 26327955

[47] Mohan A, Thachil A, Sundar G, Sastry BKS, Hasan A, Sridevi C, et al. Ventricular tachycardia and tuberculous lymphadenopathy: Sign of myocardial tuberculosis? J Am Coll Cardiol. 2015; 65(2):218–220. DOI: 10.1016/j.jacc.2014.09.087 25593066

