# Supplementary material for: Imaging and Circulating Biomarker-Defined Cardiac Pathology in Pulmonary Tuberculosis: A Systematic Review
Source: Glob Heart. 2024 Nov 8;19(1):84. doi: 10.5334/gh.1369 (PMC11545927; doi:10.5334/gh.1369)
Supplement: Supplementary File. — Appendix 1 and 2. [file gh-19-1-1369-s1.pdf]

Appendix 1 - Search Strategy

1. (exp Tomography/ or exp Tomography, X-Ray Computed/) and (cardiac or cardio* or heart or coronary or myocardial).ti,ab.
2. ((cardiac or cardio* or heart or coronary or myocardial) adj5 (magnetic resonance imag$ or MRI)).ti,ab.
3. (cardiac mr* or cmri).ti,ab.
4. ((CT adj2 coronary angiography) or (coronary adj2 CT angiography) or ctca).ti,ab.
5. Coronary Angiography/ and (compute* or ct or tomograph*).ti,ab.
6. ((compute* or ct or tomograph*) adj3 angiograph*).ti,ab.
7. ((coronary adj2 computed tomograph* angiography) or coronary cta or ccta).ti,ab.
8. exp Positron-Emission Tomography/ and (cardiac or cardio* or heart or coronary or myocardial).ti,ab.
9. ((cardiac or cardio* or heart or coronary or myocardial) and ((positron emission adj2 tomograph$) or pet or petct)).ti,ab.
10. exp Troponin/ and (cardiac or cardio* or heart or coronary or myocardial).ti,ab.
11. ((cardiac or cardio* or heart or coronary or myocardial) adj6 (biomarker* or blood test*)).mp.
12. ((cardiac or cardio* or heart or coronary or myocardial) and (troponin or brain natriuretic peptide or BNP)).mp.
13. Natriuretic Peptide, Brain/ and (cardiac or cardio* or heart or coronary or myocardial).ti,ab.
14. exp Biomarkers/ and (cardiac or cardio* or heart or coronary or myocardial).ti,ab.
15. 1 or 2 or 3 or 4 or 5 or 6 or 7 or 8 or 9 or 10 or 11 or 12 or 13 or 14
16. Tuberculosis/
17. (TB or tuberc* or MTB or EPTB or PTB).mp.
18. (TBHIV or HIV or HIVTB or human immunodeficiency virus*).mp.
19. 16 or 17 or 18
20. 15 and 19

Observational studies																	
Study	Type of study	1. Was the research question or objective in the paper clearly stated?	2. Was the study population clearly specified and defined?	3. Was the participation rate of eligible persons at least 50%?	4. Were all the subjects selected or recruited from the same or similar populations (including the same time period)? Were inclusion and exclusion criteria for being in the study prespecified and applied uniformly to all participants?	5. Was a sample size justification, power description, or variance and effect estimates provided?	6. For the analyses in this paper, were the exposure(s) of interest measured prior to the outcome(s) being measured?	7. Was the timeframe sufficient so that one could reasonably expect to see an association between exposure and outcome if it existed?	8. For exposures that can vary in amount or level, did the study examine different levels of the exposure as related to the outcome (e.g., categories of exposure, or exposure measured as continuous variable)?	9. Were the exposure measures (independent variables) clearly defined, valid, reliable, and implemented consistently across all study participants?	10. Was the exposure(s) assessed more than once over time?	11. Were the outcome measures (dependent variables) clearly defined, valid, reliable, and implemented consistently across all study participants?	12. Were the outcome assessors blinded to the exposure status of participants?	13. Was loss to follow-up after baseline 20% or less?	14. Were key potential confounding variables measured and adjusted statistically for their impact on the relationship between exposure(s) and outcome(s)?	Risk of Bias Reviewer 1	Risk of Bias Reviewer 2
Kahn 2020	Cohort	Y	Y	No	Y	N	Y	Y	N/A	Y	N/A	Y	N	N	Y	Good	Good
Patil 2023	Cohort	Y	Y	NR	Y	N	N	N	Y	Y	N/A	Y	N	N/A	N	Poor	Poor
Patel 2010	Cross-Sectional	Y	Y	Y	Y	N	N	N	N	Y	N	Y	N	N	Y	Fair	Fair
Bomanji 2020	Cross-Sectional	Y	Y	Y	Y	N	N	Y	N	Y	N	Y	N	N/A	Y	Good	Good

Table 1 - Risk of Bias using the NHLBI tool for Observational studies. Abbreviations: Y = yes; N = no; NR = not reported; N/A = not applicable.

Case series											
Study	Was the study question or objective clearly stated?	Was the study population clearly and fully described, including a case definition?	Were the cases consecutive?	Were subjects comparable?	Was the intervention clearly described?	Were the outcome measures clearly defined, valid, reliable and implemented consistently across all study participants?	Was the length of follow-up adequate?	Were the statistical methods well described?	Were the results well described?	Risk of Bias - Reviewer 1	Risk of Bias - Reviewer 2
Casas 2000	Y	Y	Y	Y	N/A	Y	Y	N	Y	Fair	Fair
Ankrah 2019	Y	Y	Y	Y	N/A	Y	N/A	N	Y	Poor	Poor
Mukasa 2022	Y	Y	Y	Y	Y	Y	N	Y	Y	Good	Good

Table 2 - Risk of Bias assessment using NHLBI tool - Case Series. Abbreviations: Y = yes; N = no; N/A = not applicable.
